# Case Report: Immune checkpoint inhibitor-associated myocarditis, myositis, and myasthenia gravis overlap syndrome with flow cytometric phenotyping before and after treatment in a patient with urothelial carcinoma

**DOI:** 10.3389/fimmu.2026.1793351

**Published:** 2026-06-15

**Authors:** Karen Gambina, Carly Tymm, Matthieu Paiola, Kurenai Tanji, John Zech, Robert Winchester, Adam Mor, Yevgeniya Gartshteyn

**Affiliations:** 1 Division of Rheumatology and Clinical Immunology, Department of Medicine, Columbia University Irving Medical Center, Columbia University, New York, NY, United States; 2 Department of Pathology and Cell Biology, Columbia University Irving Medical Center, Columbia University, New York, NY, United States; 3 Department of Radiology, Columbia University Irving Medical Center, Columbia University, New York, NY, United States

**Keywords:** case report, flow cytometry, immune checkpoint inhibitors, immune-related adverse events, myocarditis, myositis, and myasthenia gravis overlap syndrome

## Abstract

Immune checkpoint inhibitor-associated myocarditis, myositis, and myasthenia gravis overlap syndrome (IM3OS) is an uncommon immune-related adverse event (irAE) associated with the use of immune checkpoint inhibitors for the treatment of malignancies. We present a case of a 77-year-old woman who received anti-PD1 treatment for high-grade invasive urothelial carcinoma, and subsequently presented with weakness, ptosis, and dysarthria concerning for myositis, myocarditis, and myasthenia gravis overlap syndrome. She was successfully treated with high-dose glucocorticoids, mycophenolate mofetil, and intravenous immunoglobulin with resolution of presenting symptoms. To identify cellular subsets that mediated the disease flare, we performed flow cytometry analysis of peripheral blood mononuclear cells collected at baseline, at hospitalization and after treatment with high-dose glucocorticoids. We found an expansion of the memory CD8^+^ T-cell populations at the time of IM3OS presentation. Additionally, we identified a differentiated CD27^-^ CD28^-^ effector memory (EM) CD4^+^ subset that was associated with clinical disease activity and contracted with glucocorticoid use administration. This illustrates a unique case of a patient who was successfully treated for IM3OS and highlights the potential use of flow cytometry to provide insight into the underlying pathophysiology of irAEs, and to guide and gauge response to immunomodulatory therapy, thus facilitating a precision medicine approach within the diagnostic and treatment framework for irAEs.

## Introduction

Immune checkpoint inhibitor (ICI)-associated myocarditis, myositis, and myasthenia gravis overlap syndrome (IM3OS) is an immune-related adverse event related to the use of immune checkpoint inhibitors for the treatment of malignancies. ICIs block inhibitory regulatory receptors and their ligands on immune cells and tumors, thereby increasing T-cell activation, which in turn enhances T-cell-mediated immune response against the cancer. Examples of molecules targeted by ICIs include cytotoxic T-lymphocyte antigen-4 (CTLA-4), programmed cell death receptor 1 (PD-1), and programmed cell death ligand 1 (PD-L1). While ICIs have proven to be beneficial in the treatment of malignancies due to their anti-tumor effect, they can lead to unintended adverse effects, such as immune-related adverse events (irAEs). irAEs result from enhanced T-cell activation and a loss of self-tolerance, leading to non-tumor organ damage with irAE severity that can range from mild to life-threatening.

ICI-associated myocarditis, myositis, and myasthenia gravis overlap syndrome (IM3OS) is a particularly severe irAE. Patients present with concurrent myocarditis, myositis, and myasthenia gravis that can be challenging to both diagnose and treat. Specifically, IM3OS can lack features that are often seen in classical autoimmune myocarditis, myositis, and myasthenia gravis. For example, minimal to no myocyte injury, inflammation, or fibrosis may be seen on cardiac imaging or histopathologic analysis of endomyocardial tissue in ICI-associated myocarditis (1, 2). There are reported cases of ICI-associated myositis in which serum creatine kinase levels are within normal limits or in which a discordance between serum aldolase and creatine kinase levels is observed, with aldolase elevated while creatine kinase remains normal (3). Additionally, repetitive nerve stimulation and pyridostigmine/edrophonium testing can be normal in ICI-associated myasthenia gravis; notably, the positivity rates of repetitive nerve stimulation testing and serological anti-acetylcholine receptor and anti-muscle-specific kinase antibodies are lower in immune-related myasthenia gravis than in classical myasthenia gravis (4). Lastly, serologic testing can be negative in irAEs, with no autoantibodies that are classically implicated in the development of other types of autoimmune myositis or myasthenia gravis identified (5). Symptoms of IM3OS can progress rapidly and culminate in significant morbidity, including respiratory failure, cardiogenic shock, myasthenic crisis, and even death; one systematic review observed a mortality rate of approximately 60% (2). In this report we describe a case of IM3OS that highlights these challenges. We also identify immune cell subsets that are expanded in the circulation during the inflammatory phase of this disease and contract with treatment, suggesting a pathogenic role in this disease.

## Methods

Peripheral blood samples were obtained from the case patient and from five healthy individuals without cancer who served as controls. Peripheral blood mononuclear cells were isolated using Lymphoprep Density Gradient Medium (07811, STEMCELL Technologies, Vancouver, British Columbia, Canada). 2×10^6^ cells were incubated with 1:200 diluted UV Zombie (BioLegend San Diego, California, USA) in phosphate buffered saline (PBS) for 30 minutes at room temperature and protected from light. After a wash with staining buffer (PBS supplemented 2% bovine serum albumin (BSA) and 0.1% sodium azide) and centrifugation at 500 g for five minutes, cells were incubated with Fc Receptor Blocking Solution (Human TruStain FcX, BioLegend) for 10 minutes. Cells were stained with flow antibodies (see **Supplementary Table 1**) supplemented with CellBlox Blocking Buffer and Super Bright Complete Staining Buffer (Invitrogen, Waltham, Massachusetts, USA). Cells were then fixed with 4% paraformaldehyde Fixation Buffer (BioLegend #420801) and treated with Intracellular Staining Fixation Buffer (BioLegend 420801 and 421002) before intracellular staining for granzyme B and SAP. Spectral flow data was obtained on the Cytek 5 L Aurora (Cytek Biosciences, Fremont, California, USA) and analyzed in FCS Express Research V.7.

## Case presentation

**Figure 1:**
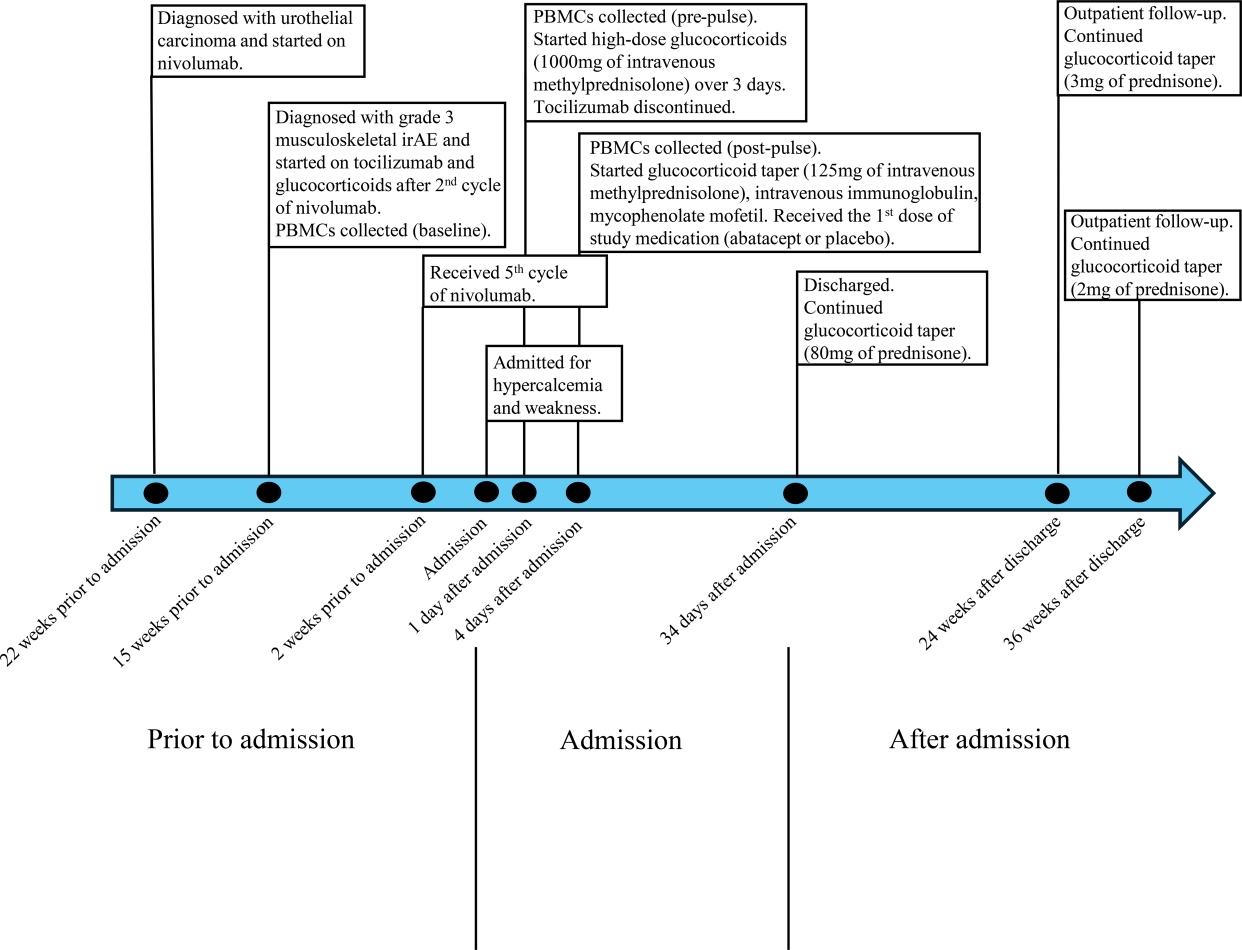
Timeline depicting order of events, including malignancy diagnosis, irAE diagnoses, hospitalization, initiation of treatment, discharge, and collection of peripheral blood mononuclear cells (PBMCs) specimens.

A 77-year-old woman with a history of type 1 diabetes mellitus and hypothyroidism was diagnosed with stage four high-grade invasive urothelial carcinoma and started treatment with nivolumab, a PD-1 antagonist. After her second cycle of nivolumab, she developed myalgias and shoulder and hip girdle stiffness. On initial evaluation at our rheumatology clinic, she was diagnosed with a grade three musculoskeletal immune-related adverse event with polymyalgia rheumatica-like features. Treatment was started with prednisone 20 mg with resolution of joint pain; the prednisone was then tapered over the next three months, and anti-IL-6 therapy with tocilizumab was initiated for the treatment of arthritis. Given her symptomatic improvement and promising response to nivolumab, the decision was made to resume immunotherapy. Peripheral blood mononuclear cells (PBMCs) were collected at the time of the initial rheumatologic evaluation, approximately 15 weeks before the subsequent hospitalization (**Figure 1**).

Several days after the fifth cycle of nivolumab, she developed weakness, arthralgias, and myalgias, followed by ptosis, dysphagia, and dysarthria two weeks later. Laboratory testing at that time revealed severe hypercalcemia to 14.6 mg/dl, and she was then admitted for further evaluation. Her physical examination on initial evaluation was notable for pronounced proximal weakness of the upper and lower extremities, ptosis of the right eyelid, and fatigability with repetitive movements. Laboratory studies revealed notable findings, including elevated blood levels of creatinine, aspartate aminotransferase, alanine aminotransferase, troponin, B-type natriuretic peptide (BNP), creatine kinase, aldolase, and C-reactive protein (**Table 1**). She was evaluated by endocrinology, who suspected nivolumab-induced hypercalcemia and recommended intravenous fluids and calcitonin, with rapid resolution of hypercalcemia.

**Table 1:** Laboratory data collected at different time points, including prior to development of IM3OS, on hospital admission and prior to administration of glucocorticoids, after administration of glucocorticoids, and several weeks after discharge from hospitalization.

Variable	Reference Range, Adults	15 Weeks before Admission	On Admission	After IV Steroid Pulse	24 Weeks after Admission	36 Weeks after Admission
Hemoglobin (g/dL)	11.2-14.7 g/dL	12.7	12.6	10.1	11.8	12.5
Hematocrit (%)	33.8043.4%	39.0	35.5	28.4	37.0	38.7
White Blood Cell Count (per µL)	3.48-9.42 x10(3)/µL	5.5	6.75	10.27	4.77	6.98
Platelets (per µL)	167-374x10(3)/µL	261	385	346	216	306
Calcium (mg/dL)	8.8-10.3 mg/dL	9.8	14.6	9.8	9.7	10.5
Creatinine (mg/dL)	0.50-0.95 mg/dL	1.25	1.53	1.47	1.21	1.21
Aspartate Aminotransferase (U/L)	9-50 U/L	82	93	113	19	15
Alanine Aminotransferase (U/L)	10-37 U/L	76	127	66	16	19
Creatine Kinase (U/L)	29-143 U/L	130	923	107	—	—
Aldolase (U/L)	≤ 8.1 U/L	—	60	15	—	—
Troponin, High Sensitivity (ng/L)	≤ 14 ng/L	—	588	130	33	51
B-type Natriuretic Peptide (pg/mL)	0.0-624.0 pg/mL	—	1484	5667	127	—
C-Reactive Protein (mg/L)	< 8.0 mg/L	10.7	19.2	—	3.6	—
Erythrocyte Sedimentation Rate (mm/hr)	1-20 mm/hr	31	28	5	—	—

**Figure 2:**
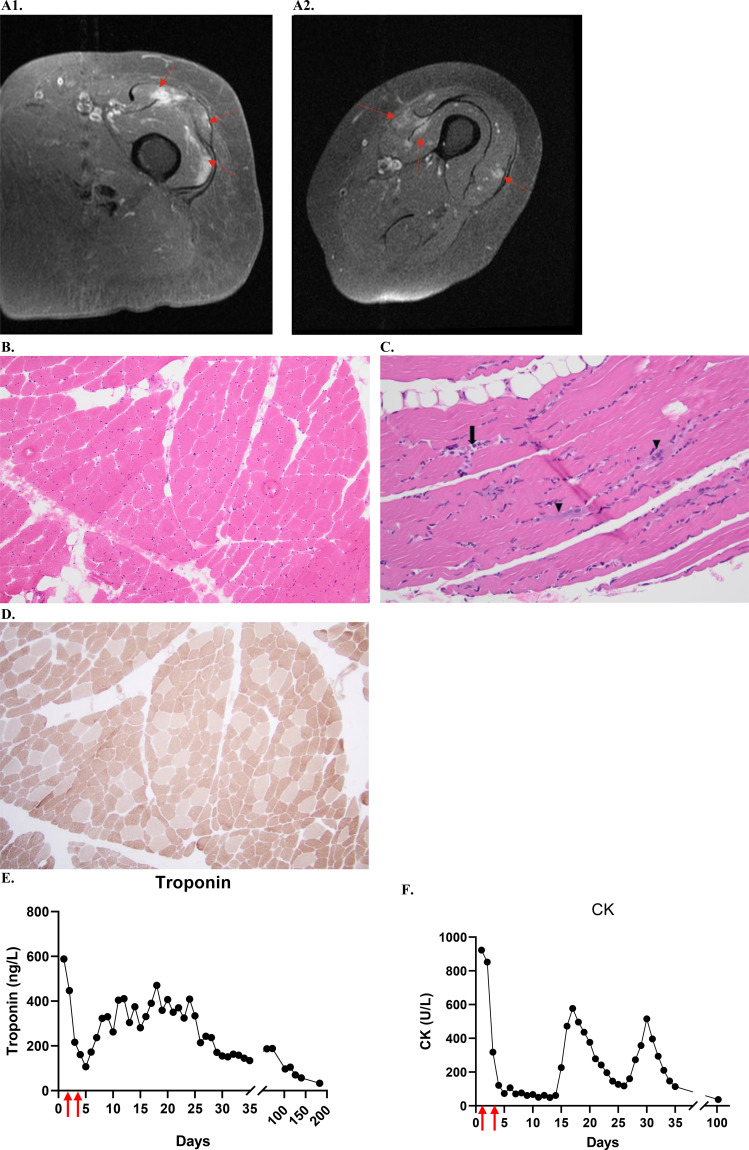
Magnetic resonance imaging of left thigh demonstrated high signal and enhancement within the anterior thigh, including within the rectus femoris muscle, vastus lateralis muscle, tensor fascia lata muscle, vastus intermedius muscle, and vastus medialis muscle on post-contrast T1 fat suppressed sequences **(A1, 2)**. Histopathological examination of left femoral quadriceps tissue (hematoxylin and eosin stain) demonstrated numerous atrophic fibers varying in size **(B)**, as well as extremely rare necrotic (arrow) and regenerating fibers (arrow heads) **(C)**. Myosin ATPase-histochemistry showed atrophic fibers, chiefly type 2 **(D)**. Sections of muscle tissue were stained with solutions containing ATP, sodium barbital, calcium chloride, and distilled water; type 1 muscle fibers are identified as pale whereas type 2 muscle fibers are dark brown. Troponin trend over the first six months following diagnosis with IM3OS, with arrows indicating time during which high-dose methylprednisolone pulse was administered. Creatine kinase (CK) trend over the first four months following diagnosis with IM3OS, with arrows indicating time during which high-dose methylprednisolone pulse was administered **(E)**. A decrease in troponin and creatine kinase levels were observed following administration of high-dose methylprednisolone pulse between day 1 and day 3 of hospitalization **(F)**.

The pronounced proximal weakness noticed on exam and elevated creatine kinase levels prompted further work-up for a myopathic process. Magnetic resonance imaging of the left thigh revealed increased fluid-sensitive signal with corresponding enhancement involving all muscle compartments of the thigh consistent with muscle edema and likely myositis (**Figure 2A**). Electromyography showed fibrillations and positive sharp waves, suggestive of an inflammatory myopathy with muscle necrosis. Biopsy of the left femoral quadriceps muscle was notable for severe type II myofiber atrophy associated with rare necrotic and regenerating fibers, rare CD3^+^ T-cells and inconspicuous CD20^+^ B-cells, as well as scattered CD68^+^ histiocytes primarily in the endomysium and the perimysial and epimysial adipose tissue (**Figures 2B–D**).

A cardiac workup revealed elevated troponin at 588 ng/L and elevated BNP at 10,264 pg/mL. Electrocardiography showed long sinus pauses and episodes of junctional escape rhythm. An echocardiogram revealed normal biventricular function, with a left ventricular ejection fraction of 60-65% and a small amount of fluid or echogenic material in the pericardial space; no segmental wall motion abnormalities were noted. Cardiac catheterization revealed normal coronary arteries, such that an ischemic etiology was deemed to be unlikely. The elevated troponin and BNP in the context of new arrhythmias raised concern for myocarditis. An endomyocardial biopsy was performed, which revealed myocardium with no significant histologic findings on light microscopy and no significant immunofluorescence reactivity.

The neurological exam was notable for skeletal muscle fatigability, ptosis, and diplopia with upward gaze, concerning for a neuromuscular junction disorder, such as myasthenia gravis. Electromyography and repetitive nerve stimulation, however, were unremarkable including no abnormal increment or decrement at rest or after exercise. Laboratory testing for myasthenia gravis-associated antibodies, including anti-acetylcholine receptor binding antibodies, anti-acetylcholine receptor modulation antibodies, anti-acetylcholine receptor blocking antibodies, anti-muscle-specific kinase antibodies, anti-voltage-gated calcium channel antibodies, and anti-low-density lipoprotein receptor-related protein four antibodies, was also negative.

Additional laboratory studies were notable for negative antinuclear antibodies, anti-Smith antibodies, anti-RNP antibodies, anti-Jo-1 antibodies, anti-Scl-70 antibodies, anti-SSA52 antibodies, anti-SSA60 antibodies, anti-SSB antibodies, anti-SAE1 antibodies, anti-NXP2 antibodies, anti-MDA5 antibodies, anti-TIF-1 gamma antibodies, anti-Mi-2 antibodies, anti-P155/140 antibodies, anti-PL-12 antibodies, anti-PL-7 antibodies, anti-OJ antibodies, anti-EJ antibodies, anti-SRP antibodies, anti-Ku antibodies, anti-Smith/RNP antibodies, anti-PM/Scl 100 antibodies, and anti-fibril antibodies (see **Supplementary Table 2**).

Her symptoms persisted despite correction of blood calcium levels. Given the clinical suspicion for IM3OS secondary to nivolumab, treatment with high-dose glucocorticoids (1000 mg of intravenous methylprednisolone) was administered for three days, followed by a slow steroid taper over the next several months. PBMCs were collected both before initiation of high-dose glucocorticoids and again after completion of three days of high-dose glucocorticoids. Additional treatment with intravenous immunoglobulin and mycophenolate mofetil was started. She was additionally enrolled in a randomized, double-blind, placebo-controlled trial of abatacept for ICI myocarditis (6) and received three doses of the study medication while hospitalized. Following the initiation of immunosuppressive therapy, ptosis and dysarthria resolved after three days, and muscle strength improved over the next few weeks, with resolution of proximal muscle weakness at the time of discharge, approximately one month after she was admitted. Troponin remained elevated for several weeks, then trended down after approximately one month of therapy and reached a near-normal nadir six months later. Serum levels of creatinine, aspartate aminotransferase, alanine aminotransferase, creatine kinase, aldolase, BNP, and C-reactive protein normalized following treatment (**Figures 2E, F**).

She was admitted for a total of 34 days before discharge. Nivolumab was discontinued, and cancer-directed therapy was withheld until a repeat PET-CT performed approximately three months after discharge demonstrated new FDG-avid abdominopelvic lymph nodes concerning for new metastases. There was no recurrence of hypercalcemia. She was continued on mycophenolate mofetil and prednisone for several months. The decision to re-challenge the patient on an ICI was ultimately deferred.

**Figure 3:**
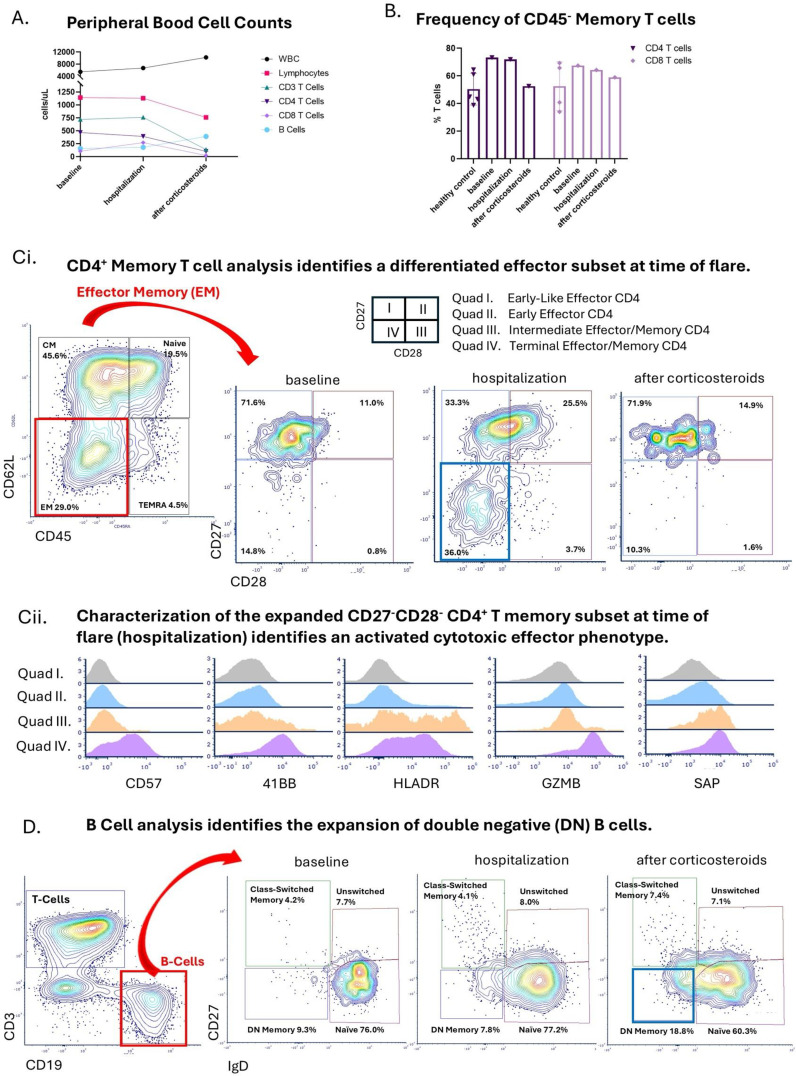
Flow cytometric phenotyping of peripheral blood mononuclear cells that were acquired from blood specimens collected 15 weeks prior to the patient’s hospitalization (which served as a baseline), on hospital admission but prior to administration of high-dose glucocorticoids, and 3 days after administration of high-dose glucocorticoids were completed. Cell counts were derived from an automated CBC analysis that provided the absolute counts of WBC and Lymphocytes per uL. The counts of CD3, CD4, CD8 and B cells were calculated from the percentage of lymphocytes based on flow cytometry staining of PBMCs isolated on that same day. Following high-dose glucocorticoid treatment, there was a significant decrease in total CD3^+^ T cell counts, with similar trends observed in the CD4 and CD8 subsets **(A)**. The proportion of CD4^+^ and CD8^+^ CD45RA-memory subsets at baseline and at the time of hospitalization as compared with four healthy controls is shown. **(B)**. Effector memory cells (CD45RA^-^, CD62L^-^) were further evaluated based on CD27 and CD28 expression **(Ci)**. The CD27^-^CD28^-^ subset, expanded at time of hospitalization, was further analyzed for the expression of markers of activation and cytotoxicity as shown. **(Cii)**. CD19^+^ B Cells were analyzed based on IgD and CD27 expression, with expansion of the double negative (DN) subset seen after glucocorticoid administration, compared to both baseline and disease flare states **(D)**.

We used flow cytometry to analyze the PBMCs collected 15 weeks prior to hospitalization (serving as a baseline), disease flare on hospital admission but prior to glucocorticoids, and three days after administration of high-dose glucocorticoids (see **Supplementary Figure 1** for flow gating strategy). At the time of disease flare, there was a greater than 2-fold increase in the CD8^+^ T-cell population from 108 cells/uL at baseline to 273 cells/uL at hospitalization. Following high-dose glucocorticoid treatment, there was a significant decrease in total CD3^+^ T cell counts, with similar trends observed in the CD4 and CD8 subsets (**Figure 3A**). Interestingly, the B-cell population did not undergo a similar contraction. We next compared the T cell maturation subsets (based on CD62L and CD45RA expression) across the three time-points. We found an increased proportion of CD4^+^ and CD8^+^ CD45RA^-^ memory subsets at baseline and at the time of hospitalization (as compared with five healthy controls), which was reduced but not normalized following glucocorticoid administration (**Figure 3B**).

The maturation state of memory CD4^+^ T cells can be further differentiated based on CD27 and CD28 expression. We further analyzed the CD45RA^-^CD62L^-^ Effector Memory (EM) subset and identified early, intermediate, and terminal effector subsets based on loss of CD27 and CD28 expression (**Figure 3C**). We found that at the time of hospitalization, there was an increase in Intermediate and Terminal Effector subsets, which were markedly reduced following high-dose glucocorticoid administration (**Figure 3Ci**). A deeper characterization of these subsets revealed increased expression of activation markers, such as 4-1BB, HLA-DR, and SAP, as well as terminal differentiation and cytotoxicity markers, including CD57 and GZMB (**Figure 3Cii**; see **Supplementary Figure 2** for expression of activation markers across all three timepoints). We thus found that within the memory CD4^+^ T cells, an increase in the terminally differentiated subsets corresponded to clinical disease flare and further contracted with glucocorticoid treatment and symptom improvement. This subset was marked by expression of activation and terminal differentiation markers with cytotoxic potential.

Regarding B cell populations, there were minimal changes in the memory B cell subsets that correlated with disease flare (**Figure 3D**). However, we observed an expansion of the double-negative (DN) IgD^-^ and CD27^-^ B cell population after glucocorticoid administration, compared to both baseline and disease flare states.

## Discussion

Here we present a case of a patient with urothelial carcinoma on nivolumab who developed IM3OS, which was successfully treated with high-dose glucocorticoids, intravenous immunoglobulin, and mycophenolate mofetil. Flow cytometric phenotyping of peripheral blood mononuclear cells derived from blood specimens collected at three different time points throughout the patient’s disease course was also carried out to further characterize the patient’s lymphocyte populations before and after treatment.

This case is unique for several reasons. First, we highlight the diagnostic challenge posed by IM3OS, including the extensive serologic, radiographic, and histopathologic work-up required, and the critical role of clinical suspicion and thorough physical examination, particularly when diagnostic testing is negative or inconclusive. Second, we describe the case of a patient who was successfully treated for IM3OS. Despite the high mortality associated with this condition, which can approximate 46% in ICI myocarditis alone and 60% in IM3OS, our patient was discharged from the hospital, returned to a baseline level of function in the outpatient setting, and was able to pursue eventual tapering of immunosuppressive therapy and continuation of cancer-directed therapy (2, 7).

Using flow cytometry, we characterized the serial changes in the circulating mononuclear cell subsets seen in our patient with clinically suspected IM3OS. At the time of hospitalization, we identified an expansion of effector memory T-cell populations, similar to what has been reported by others. Specifically, Zhu et al. found an expansion of cytotoxic CD8^+^ T-cells in patients with ICI myocarditis that were not significantly reduced following treatment with glucocorticoids, suggesting the persistence of aberrantly expanded cell populations such as the terminally differentiated TEMRA CD8^+^ T cells (8). Similar to the observation by Zhu et al., we did not find a significant reduction in the TEMRA T-cell population following treatment with glucocorticoids. However, we identified an expanded population of differentiated effector memory CD4^+^CD27^-^CD28^-^ T cells with increased expression of activity markers, such as 4-1BB and HLA-DR, as well as GZMB^+^ cytotoxic potential. Following treatment with high-dose glucocorticoids, we noted a near-complete eradication of this cluster. The identification of this activated effector subset with cytotoxic potential, correlating with disease activity, suggests that this subset may have had a role in driving the development of IM3OS in our patient. The ensuing contraction of these cell populations following treatment suggests that high-dose glucocorticoids were effective in depleting the aberrant cell subpopulations and achieving a lymphocyte distribution that approximated the baseline level before the development of IM3OS. Interestingly, we also observed an expansion of the double-negative (DN) B-cell population following treatment with high-dose glucocorticoids that was not present at baseline nor at the time of IM3OS development. DN cells, which are increased in a range of autoimmune diseases, including systemic lupus nephritis, rheumatoid arthritis, and myasthenia gravis, can express high levels of cytokine receptors in response to toll-like receptor stimulation and have the potential to differentiate into antibody-secreting plasma cells (9, 10). However, the functional significance and mechanistic role of these DN B cells in irAE pathogenesis remain unclear and warrant further investigation.

The strength of our approach in this case lies in our use of flow cytometric analysis to identify changes in lymphocyte populations throughout this patient’s disease course. It highlights the potential of flow cytometry to identify dysregulated immune cell populations that may serve as a marker for identifying patients at risk of developing an irAE in the future. The early identification of patients at high risk for developing an irAE may allow for prompt intervention, which in turn may reduce morbidity and mortality, thereby improving irAE outcomes. It also raises the question of whether flow cytometry can be used to guide the choice of therapy for treating irAEs by allowing providers to select immunomodulatory agents intended to target specific lymphocyte subsets identified on flow cytometric analysis which are thought to be abnormal and involved in the development of an irAE. This may allow for the incorporation of a precision medicine approach within the diagnostic and treatment framework for irAEs. Lastly, flow cytometry can provide invaluable insight into not only the pathophysiology underlying irAEs, but also shed light on the potential long-term effects of the therapies used to treat irAEs.

One limitation of this case is that while the patient experienced a favorable outcome, we cannot comment on the generalizability of the treatment regimen for other patients diagnosed with IM3OS. Additionally, flow cytometric phenotyping was performed on blood specimens that were collected at only three discrete time points within this patient’s disease course, and we do not have additional blood specimens collected to assess for further changes in lymphocyte populations following the administration of intravenous immunoglobulin and mycophenolate mofetil, and the completion of the glucocorticoid taper. Lastly, the absence of myasthenia gravis-associated antibodies, and normal electromyography and repetitive nerve stimulation testing make it difficult to confirm the presence of ICI-related myasthenia gravis, although clinical symptomatology was suggestive of it.

## Conclusion

Immune checkpoint inhibitor-associated myocarditis, myositis, and myasthenia gravis overlap syndrome is considered a particularly severe irAE, and it remains challenging to both diagnose and treat. Flow cytometric analysis of peripheral blood mononuclear cells can provide valuable insight into the pathophysiology underlying the development of irAEs, such as IM3OS, and has the potential to become a tool for identifying patients on ICI therapy who may be at risk of developing an irAE. It may also facilitate a personalized approach to treating irAEs by guiding the selection of immunomodulatory therapy and allowing providers to gauge treatment response through subsequent alterations in lymphocyte profiles. This case illustrates the potential of flow cytometric analysis to revolutionize our diagnostic and therapeutic approaches to irAEs and may improve outcomes when incorporated into a precision medicine model for both oncologic and rheumatologic patient care.

## Data Availability

The raw data supporting the conclusions of this article will be made available by the authors, without undue reservation.

## Supplementary material

Supplementary Figure 1 Flow gating strategy utilized.

Supplementary Figure 2 Activation markers expression across the three separate timepoints.

Supplementary Table 1 Antibodies and reagents for flow cytometry.

Supplementary Table 2 Antibody test results.
