# Supplementary material for: Case Report: Immune checkpoint inhibitor-associated myocarditis, myositis, and myasthenia gravis overlap syndrome with flow cytometric phenotyping before and after treatment in a patient with urothelial carcinoma
Source: Front Immunol. 2026 Jun 15;17:1793351. doi: 10.3389/fimmu.2026.1793351 (PMC13310926; doi:10.3389/fimmu.2026.1793351)
Supplement: Supplementary Figure 1 — Flow gating strategy utilized. [file SupplementaryFile1.pptx]

## Slide 1
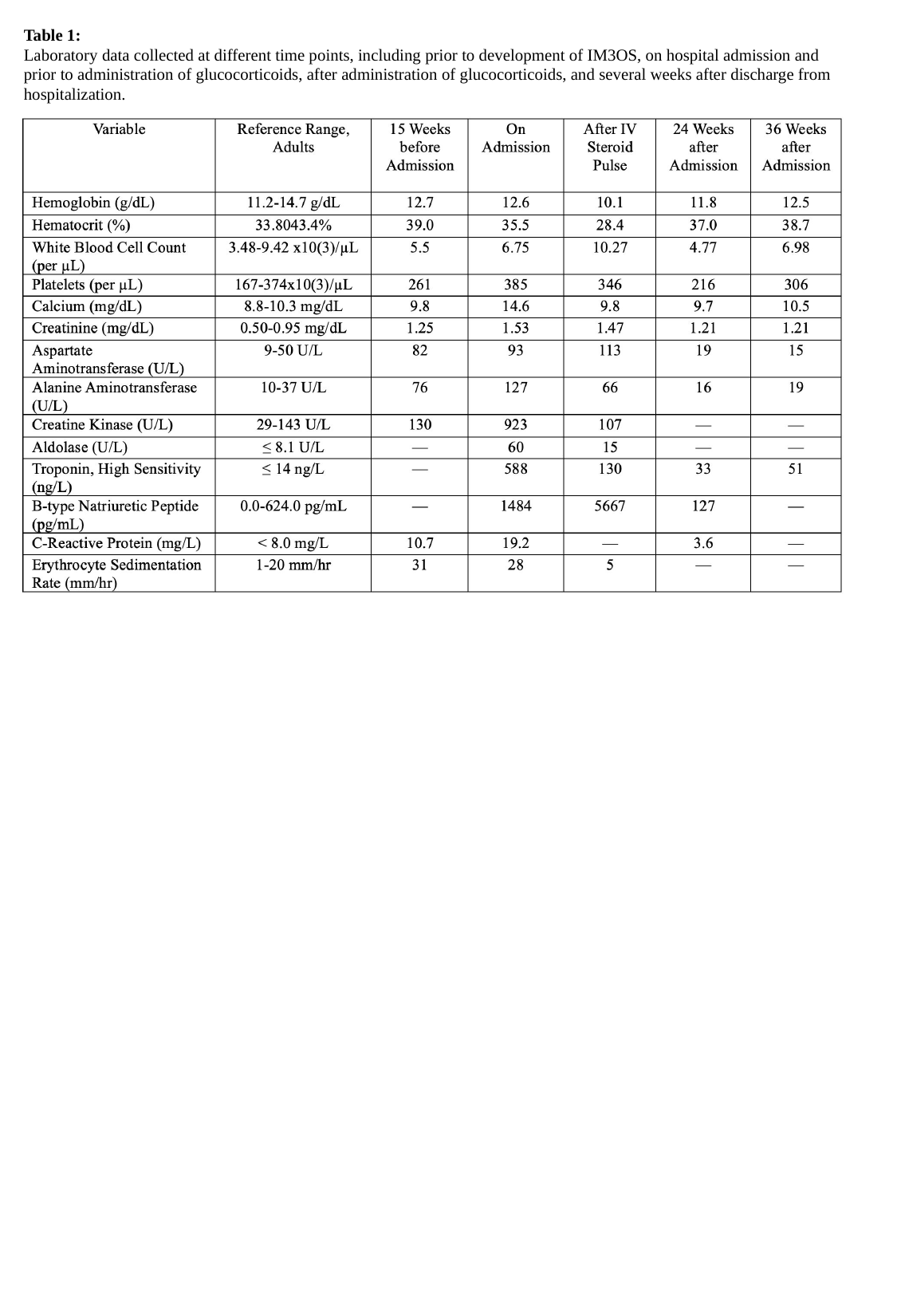

Table 1:
Laboratory data collected at different time points, including prior to development of IM3OS, on hospital admission and prior to administration of glucocorticoids, after administration of glucocorticoids, and several weeks after discharge from hospitalization.

## Slide 2
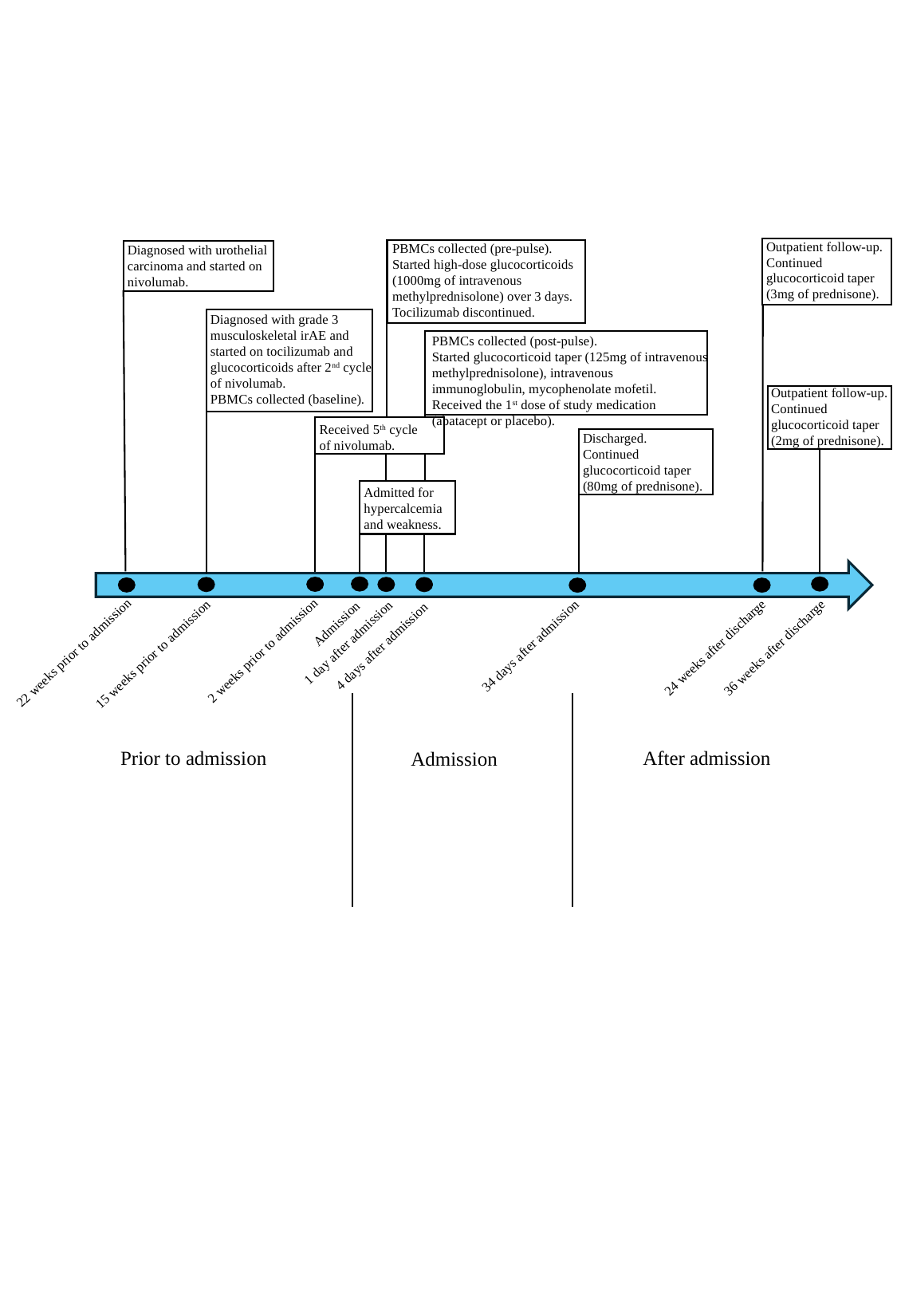

Outpatient follow-up.
Continued glucocorticoid taper (3mg of prednisone).
PBMCs collected (pre-pulse).
Started high-dose glucocorticoids (1000mg of intravenous methylprednisolone) over 3 days. Tocilizumab discontinued.
Diagnosed with urothelial carcinoma and started on nivolumab.
Diagnosed with grade 3 musculoskeletal irAE and started on tocilizumab and glucocorticoids after 2nd cycle of nivolumab.
PBMCs collected (baseline).
PBMCs collected (post-pulse).
Started glucocorticoid taper (125mg of intravenous methylprednisolone), intravenous immunoglobulin, mycophenolate mofetil. Received the 1st dose of study medication (abatacept or placebo).
Outpatient follow-up.
Continued glucocorticoid taper (2mg of prednisone).
Received 5th cycle of nivolumab.
Discharged.
Continued glucocorticoid taper (80mg of prednisone).
Admitted for hypercalcemia and weakness.
1 day after admission
4 days after admission
Admission
34 days after admission
24 weeks after discharge
36 weeks after discharge
15 weeks prior to admission
22 weeks prior to admission
2 weeks prior to admission
Prior to admission
After admission
Admission

## Slide 3
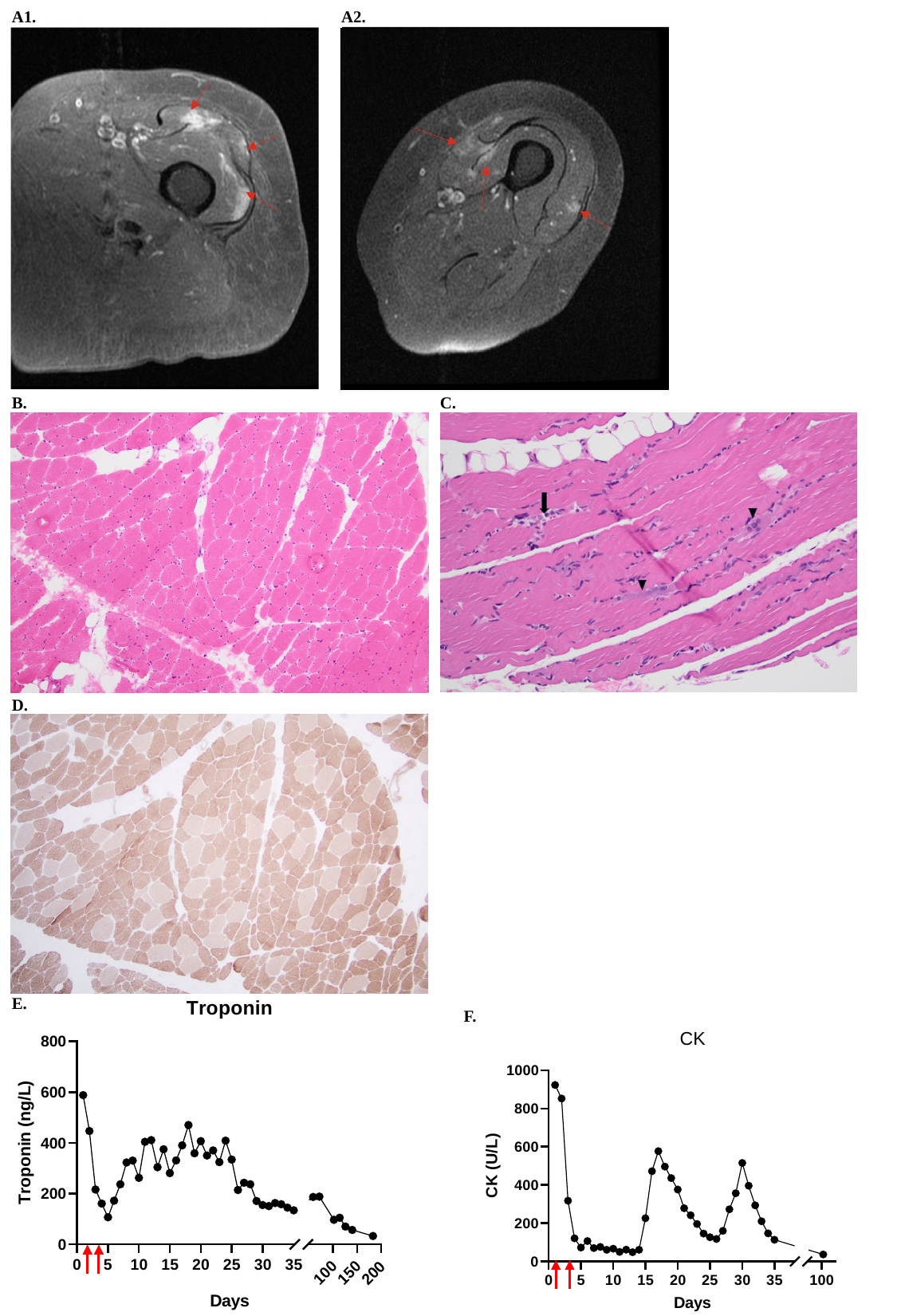

A1.
A2.
B.
C.
D.
E.
F.

## Slide 4
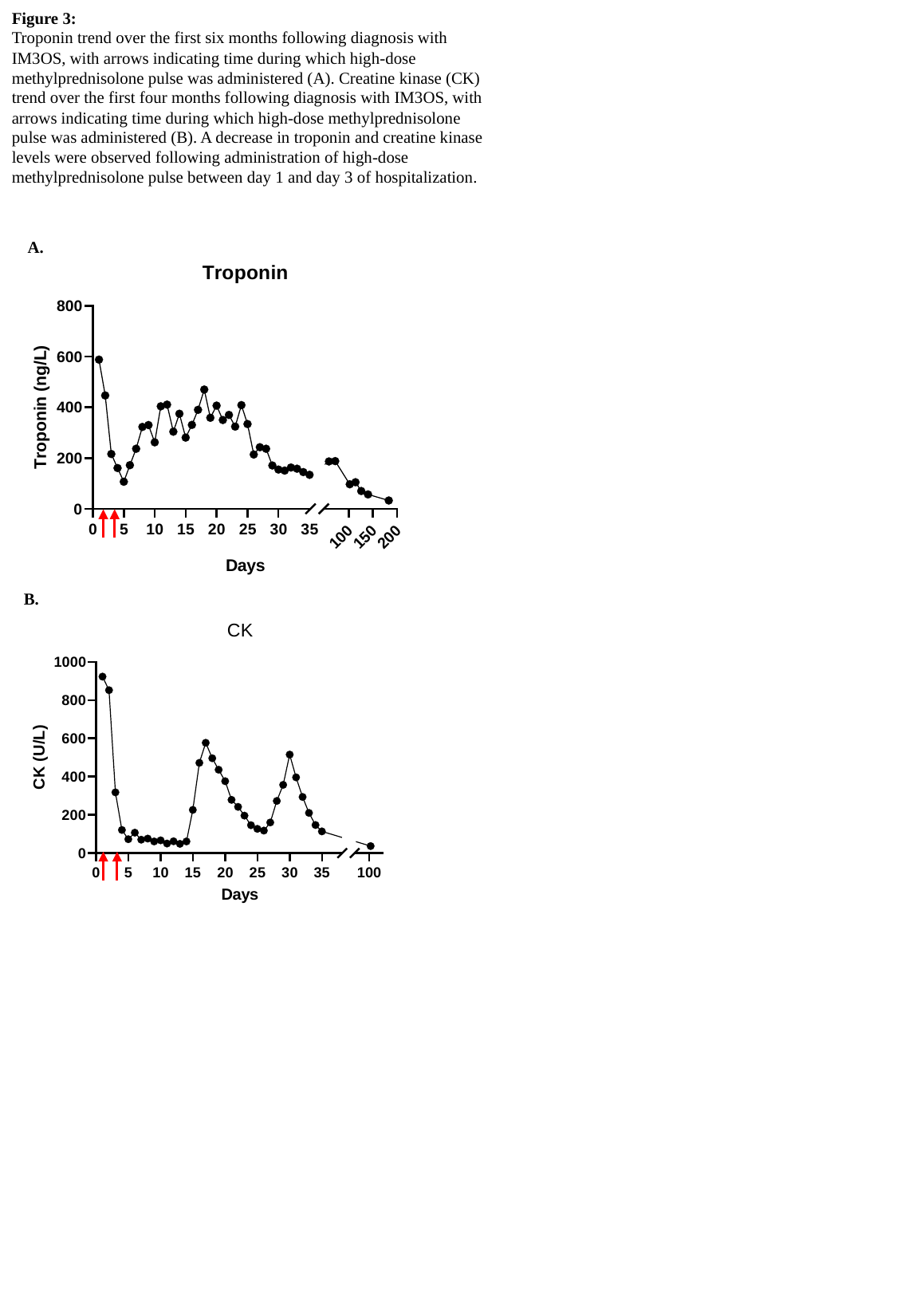

Figure 3:
Troponin trend over the first six months following diagnosis with IM3OS, with arrows indicating time during which high-dose methylprednisolone pulse was administered (A). Creatine kinase (CK) trend over the first four months following diagnosis with IM3OS, with arrows indicating time during which high-dose methylprednisolone pulse was administered (B). A decrease in troponin and creatine kinase levels were observed following administration of high-dose methylprednisolone pulse between day 1 and day 3 of hospitalization.
A.
B.

## Slide 5
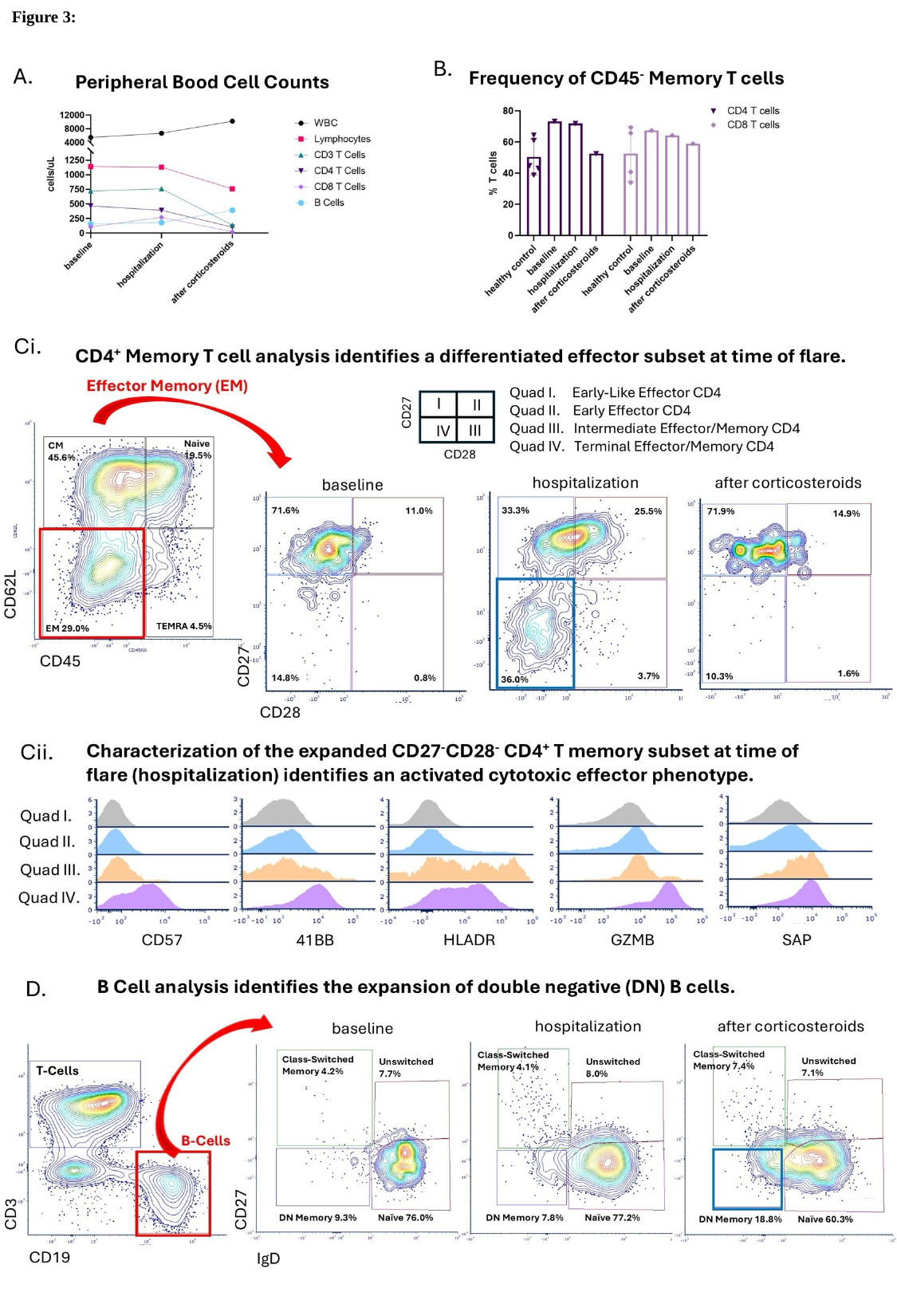

Figure 3:

## Slide 6
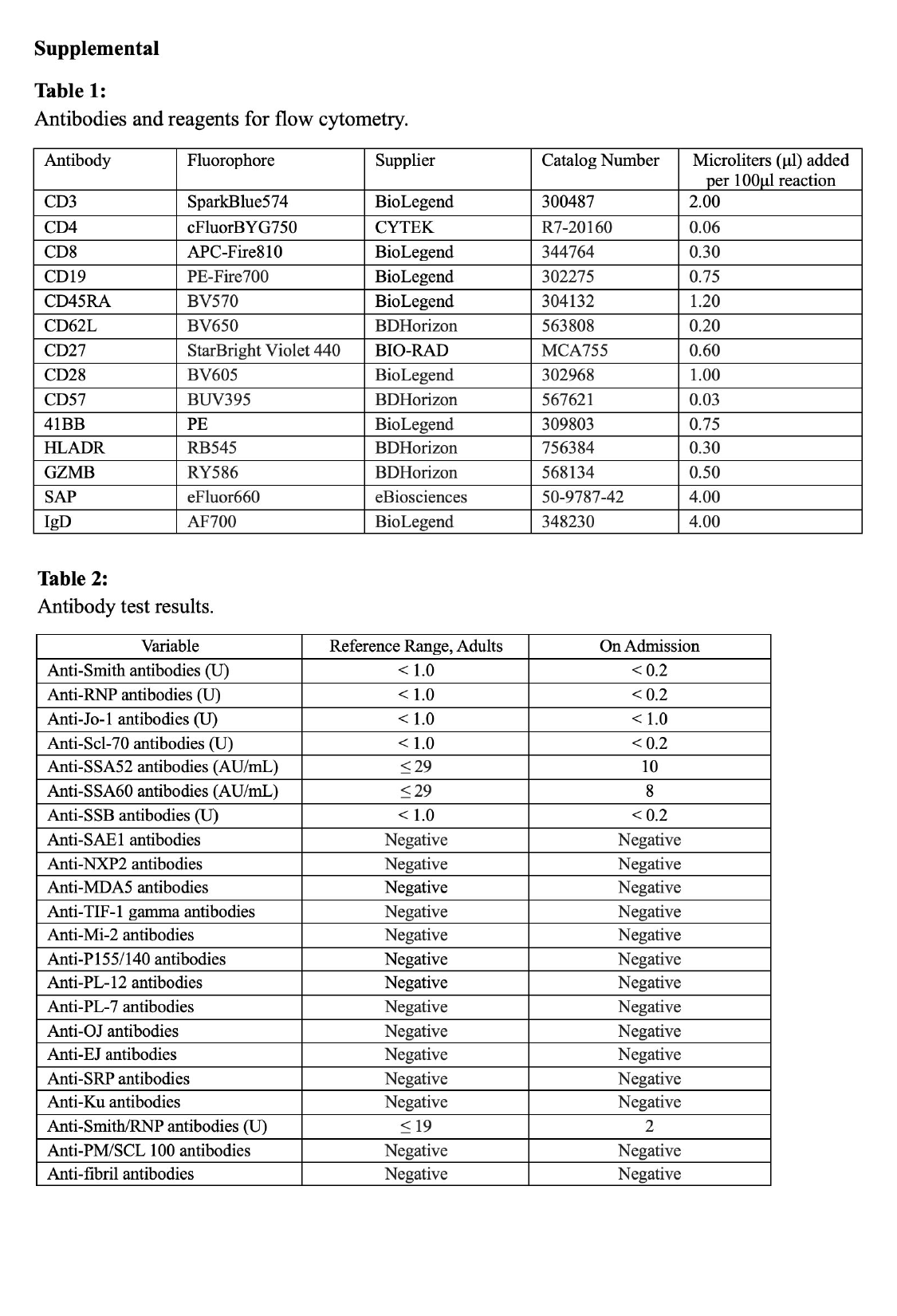

## Slide 7
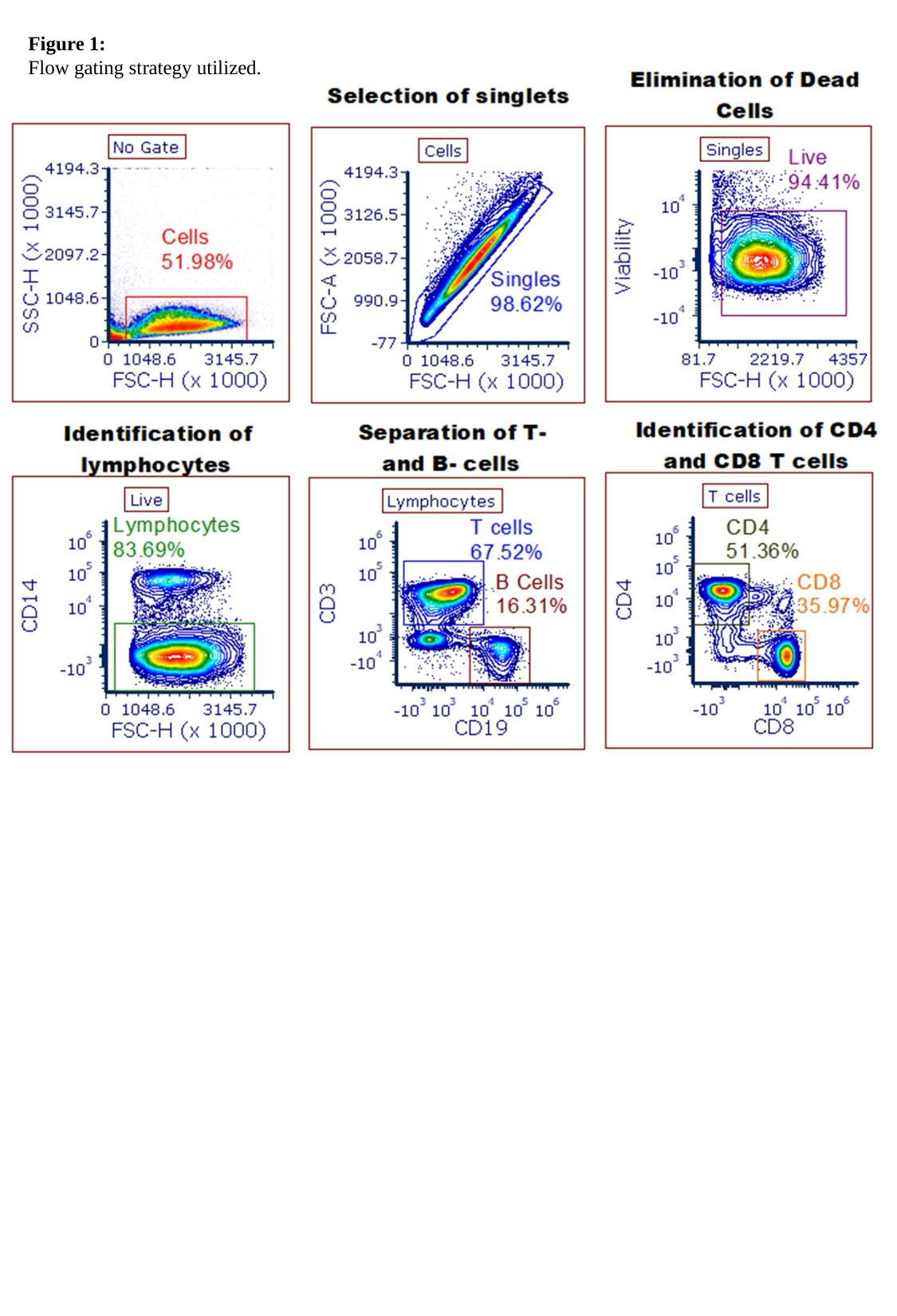

Figure 1:
Flow gating strategy utilized.

## Slide 8
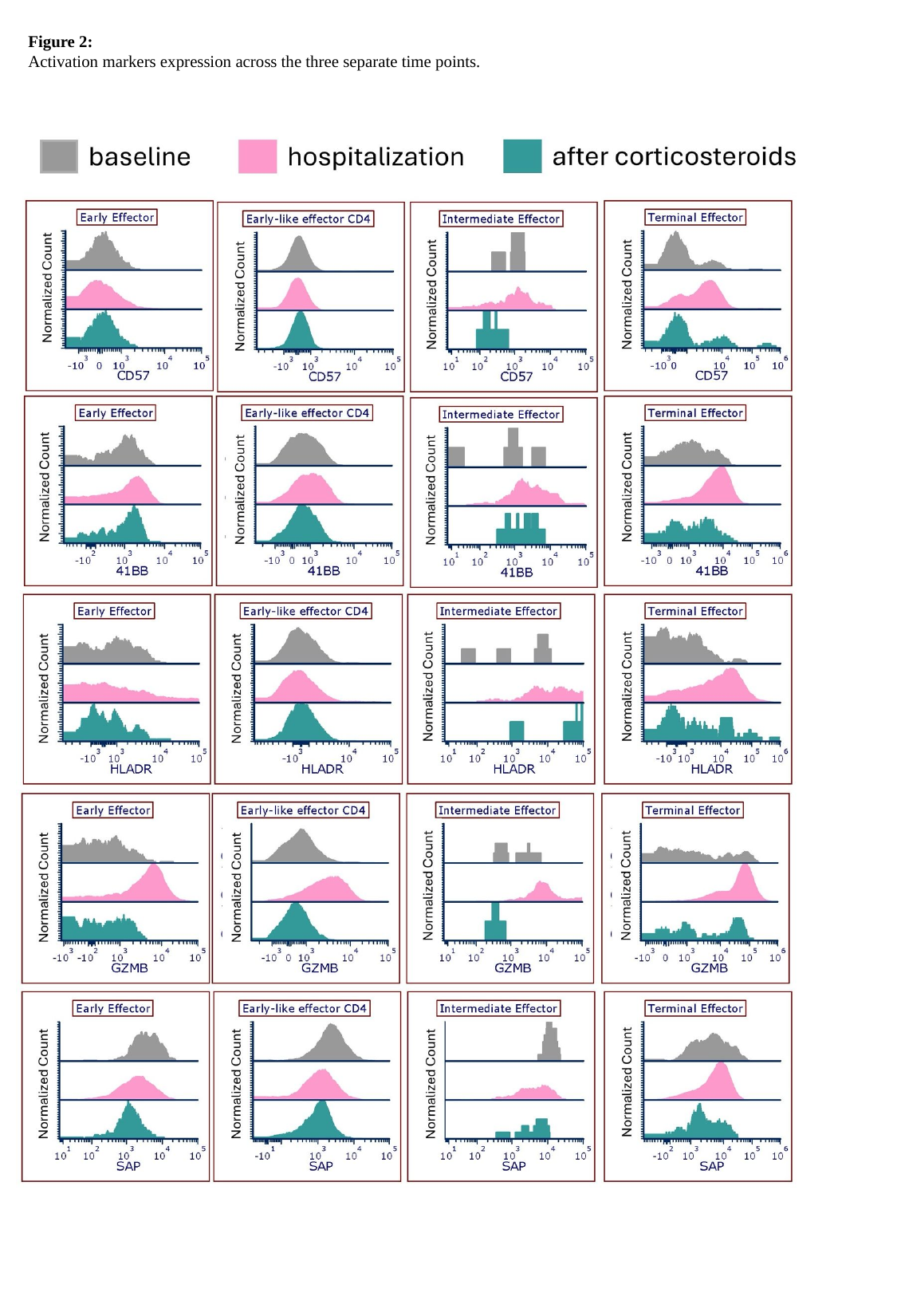

Figure 2:
Activation markers expression across the three separate time points.
